# Leveraging Spatial Transcriptomics to Decode Craniofacial Development

**DOI:** 10.3390/genes16050557

**Published:** 2025-05-03

**Authors:** Jeremie Oliver Piña, Resmi Raju, Aye Chan Myo, Evan Stipano, Malachi Wright, Rena N. D’Souza

**Affiliations:** 1 Section on Craniofacial Genetic Disorders, *Eunice Kennedy Shriver* National Institute of Child Health and Human Development (NICHD), National Institutes of Health (NIH), Bethesda, MD 20892, USA; resmi.raju@nih.gov (R.R.); ayechan.myo@nih.gov (A.C.M.);; 2 National Institute of Dental and Craniofacial Research (NIDCR), National Institutes of Health (NIH), Bethesda, MD 20892, USA

**Keywords:** spatial transcriptomics, palate, tooth, gene expression, Wnt gene signaling, patterning, cell differentiation

## Abstract

Understanding how intricate cellular networks and signaling pathways communicate during the formation of craniofacial tissues like the palate and tooth has been the subject of intense investigation for several decades. Both organ systems undergo patterning morphogenesis and the subsequent terminal differentiation of matrix-producing cells that form biomineralized matrices like bone, enamel, dentin, and cementum. Until recently, gene expression profiles could only be assessed for a select number of cells without the context of the entire milieu of genes expressed by neighboring cells and tissues. Today, the cutting-edge field of spatial transcriptomics offers a remarkable suite of innovative technologies of multiplex gene analyses and imaging that can assess the expression of a vast library of genes that are present in situ during normal and abnormal conditions. In this review, we summarize some key technologies which have in recent years enabled an unprecedented breadth and depth of transcriptomic analyses in craniofacial development. We focus in detail on select methods that our research group has applied to better understand the cellular and molecular events that drive palate and tooth development. Our overall goal is to unravel the complexities of these unique biological systems to provide meaningful biological insights into the cellular and molecular events that drive normal development. As a work-in-progress, we strive for a deeper understanding of the temporal and spatial gene expression profiles within cells and tissues during normal and abnormal palate and tooth development. Such knowledge provides the framework for further studies that can characterize the function of new or novel genes that have the potential of serving as therapeutic targets for correcting disorders like cleft palate and tooth agenesis.

## 1. Introduction

The human craniofacial complex is an exquisitely patterned system where the fusion of sutures of the cranium, face, and palate is precisely orchestrated during development. The process of cranial neural crest cell migration and growth is driven by a programmed series of cell–cell and cell–matrix interactions that involve morphogenetic gradients of signaling molecules. The importance of patterning morphogenesis is best underscored in forming tooth organs where the position, size, and shape of teeth follow antero-posterior, mesio-distal, and buccolingual gradients. It is only after morphogenesis is achieved through the collective size that distinct subpopulations of cells can be released to become specialized matrix-producing cells. The physiological function of these specialized or terminally differentiated cells correlated closely with their spatial distribution and relationship with other cells within an intact tissue or organ system.

In this review, we describe the use of unbiased approaches of spatial transcriptomics to gain a deeper understanding of the heterogeneity of cellular and molecular events that drive key events during palatal and tooth morphogenesis, as well as the terminal differentiation of osteogenic cells in the craniofacial skeleton. We also provide examples that illustrate the power of evaluating multiple cell populations in their original spatial context and in key stages in palate and tooth development.

## 2. Single-Cell RNA Sequencing, Imaging, and Spatial Analytics

As a cutting-edge technology, spatial transcriptomics is transforming modern biology and offers craniofacial biologists unprecedented opportunities to unravel the intricate actions and interactions of cells within their specific microenvironments [1]. The field has evolved rapidly from the initial application of RNA-seq analysis to single cells (scRNA-seq) to bulk populations alongside the generation of sophisticated data analytics. The availability of multiple commercialized platforms can be confusing to newcomers who have been conditioned to more bias and approaches that have relied on incremental techniques focused on individual genes and select cell populations.

As detailed in the literature, scRNA-seq continues to serve as the primary approach for deciphering the heterogeneity and complexity of RNA transcripts within individual cells, in addition to revealing the composition of numerous cell types and functions within highly organized tissues/organs/organisms [2]. Conventional transcriptomes, proteomes, or epigenomes from DNA samples and bulk RNA/DNA samples were only capable of capturing signals from tissue/organs, which fail to distinguish individual cell variations [3]. With the implementation of scRNA-seq, it is now possible to analyze gene expression profiles of individual cells rather than bulk populations of cells by dissociating cells and sequencing their RNA individually.

**Figure 1:**
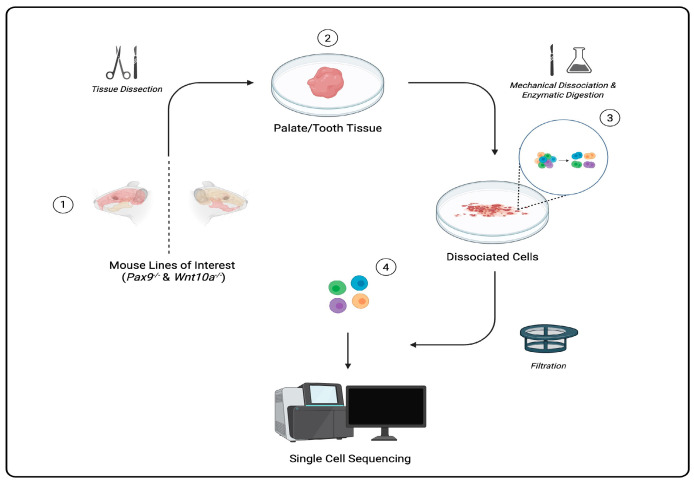
Schematic of representative workflow for tissue capture and processing for single-cell transcriptomic analysis (Created in BioRender. Piña, et al. (2025)).

Single cells can be captured through a regimented protocol involving multiple precision-driven steps: microdissecting tissue samples, isolating/dissociating cells from tissue samples, sorting each cell using fluorescently labeled antibodies that bind to specific surface proteins and markers in fluorescence-activated cell sorting (FACS), and barcoding and amplification of complementary DNA (cDNA) and library preparation [4]. scRNA-seq data are then processed and visualized to aid in the classification and identification of gene expression in cells of a heterogeneous population (Figure 1). Among many applications, scRNA-seq technology is used to cultivate atlases (catalogs of cells in all living organisms), serving as key resources to better understand cell diversity, gene expression profiles, cell development, and potential solutions for diagnosing and treating diseases [2].

As whole transcriptome data analysis tools have expanded, several key limitations of the scRNA-seq output have been identified. Given the technique sensitivity of these assays, each step in the regimented protocol can potentially give way to mixed results following downstream analysis. Specifically, the low efficiency and coverage of RNA transcript capturing may lead to loss of gene expression information (e.g., dropout), as well as batch effect implications and tissue handling protocol variation. Such heterogeneous practices can allow for contaminating signals, sometimes misidentifying cell subpopulations or genetic characteristics if proper mitigation strategies are not implemented [5]. Available computational batch correction resources such as Harmony, LIGER, Seurat v5, and MNN can largely achieve the batch integration and removal of batch effects, substantially alleviating traditional barriers to integrating datasets [6,7,8]. Furthermore, the evolution of spatial transcriptomics technology has now allowed gene expression information to be obtained from intact tissue sections in their original physiological context at a spatial resolution unlike in scRNA-seq [4,9].

Spatial RNA sequencing (spRNA-seq) technology allows for the measurement of gene expression while preserving the spatial context within tissue sections. It achieves this combined output by coupling high-throughput transcriptome data from scRNA-seq principles with the spatial resolution of high-magnification imaging approaches. While bulk and scRNA-seq approaches provide meaningful broad-coverage and temporal transcriptomic data, these techniques lack tissue-specific spatial information critical to inferring genetic regulatory networks in situ. Although some in vivo validation can occur through the lens of in situ hybridization genetic analysis, this remains primarily a qualitative validation assay.

To curtail some of these risks and limitations that are inherent to scRNA-seq approaches, spRNA-seq now enables the real-time assessment of in situ gene expression at a whole transcriptome level, generating a more preserved and complete picture of spatiotemporal gene expression. While the per-cell resolution is lower than the scRNA-seq workflow, and the two-dimensional nature of histology limits the overall cell capture, the spatial biological insight from sequencing relatively undisturbed tissue slices in situ brings higher confidence in discerning spatiotemporal gene expression trends [4].

By sectioning tissue slices (~4–8 microns) directly on glass slides for spatially resolved RNA analysis at high throughput, researchers are now able to localize gene expression within intact tissue sections to study spatial gene expression, tissue architecture, and cell–cell interactions at a much higher scale than previously possible. This enables a deeper understanding of how gene expression varies across different tissue regions, how cells communicate, and how spatial organization influences biological functions and disease progression [10]. Such technologies continue to expand into higher resolution and a greater breadth of coverage of transcriptomic targets, rapidly enhancing the level of spatial biological insights possible in preclinical and clinical tissues.

To complement and further enhance such advances in spatial analysis of transcriptomes, several imaging techniques have been developed. One example is fluorescent in situ hybridization (FISH), a technique that incorporates fluorophore-coupled nucleotides as probes to examine the presence or absence of complementary sequences in fixed cells or tissues under a fluorescent microscope. Used in various fields, FISH enables studies of gene expression, chromosomal abnormalities, and DNA–protein interactions by using probes specific for short-strand DNA. In the application of FISH, we begin with sample preparation including any pre-hybridization steps that may be required, the addition and hybridization of probes to the desired target DNA sequence, washing unbound probes, and the visualization of results, which must be conducted to successfully utilize this technique. The implementation of this technique has been monumental in the detection of infectious diseases and single-cell DNA. It also serves as an adjunctive diagnostic tool for both constitutional and somatic cytogenetic abnormalities [11].

Like FISH, one specific technological advancement in applied spatial analysis is an RNA-ISH method, RNAscope (ACD, Biotechne, Inc., Newark, CA, USA). RNAscope is commonly used in various fields like cancer research, developmental biology, and infectious disease to study gene expression patterns and to better understand how RNA dynamics contribute to cellular function and disease processes. This method uses a double Z probe design with adjacent probes that bind to the target-specific site, allowing for the detection of specific RNA sequences directly in tissue sections or cells with high spatial resolution. This method is mainly used for the single-molecule-level detection of mRNA in situ, enabling spatial transcriptomics, gene expression profiling, and the detection of low-expression genes in tissues. Chromogenic (brightfield) or fluorescent signals allow us to detect RNAscope data visually. Open-source methods (SMART-Q, dotdotdot, HALO, etc.) are available for quantifying fluorescence-amplification-based RNAscope in situ hybridization signals [12].

More recent developments in genome research includes techniques driven by machine learning and other artificial intelligence (AI) principles, allowing for greater integration across omics datasets. Traditional microarray data analysis relies on statistical methods for pattern recognition, classification, and biomarker discovery. The implementation of AI-driven analytical tools in genomics will continue to aid researchers in analyzing data efficiently and allowing for the clinical implementation of non-invasive real-time disease biomarker monitoring and therapeutic discovery [13].

## 3. Spatial Transcriptomics to Study the Developing Palate

When disrupted, the dynamic morphogenetic processes required for proper development of the palate—an anatomic bridge separating the oral and nasal cavities—can manifest as a palatal cleft (a subset of orofacial clefts). Despite the prevalence of palatal clefts in humans, occurring in approximately 1 out of every 700 live births, the pathophysiology is complex and remains incompletely understood. Among the leading hypotheses, our research group has focused primarily on investigating disruptions in the Pax9-driven Wnt signaling pathway. Along with other groups, we have shown that the exclusive molecular relationship of Pax9 with Wnt genes is critical for the patterning and osteogenic differentiation that leads to growth and fusion of the posterior palatal shelves. Disruptions at key developmental timepoints associated with embryonic fusion of the palatal shelves account for the formation of cleft palate anomalies.

Prior to the advent of spatially resolved gene expression analysis, or “spatial transcriptomics”, exploration of the genomic basis of palatal clefts was limited to traditional RNA sequencing (RNA-seq) studies—such as bulk and single-nucleus RNA-seq. While these approaches are valuable in that they can provide meaningful global temporal transcriptomic data, they fail to offer tissue-specific, spatial information critical to inferring genetic regulatory networks involved in palatal clefting in vivo. Thus, while traditional methods of RNA sequencing analyses remain an important component of our cleft studies, the coincident emergence of spatial transcriptomics and new, more powerful bioinformatics pipelines presents an unprecedented platform for studying the palate at high spatial resolution.

Using unbiased approaches, our group has investigated palatal development using several different spatial transcriptomic approaches. Principally, Visium/Visium HD and Xenium In Situ (10X Genomics, Inc., Pleasanton, CA, USA) have been the cornerstone of our spatial analyses. Visium, and its subsequent iteration, Visium HD (commercially available since March 2024), leverages a next-generation-sequencing-based approach that combines single-cell-level RNA sequencing with spatially resolved transcript localization. In our Visium/Visium HD experiments, the platform’s per-cell biological insight allowed us to perform conventional single-cell analyses. When coupled with spatiotemporal gene expression analyses, we have opened new avenues of discovery in palatal development. For example, in the study published in 2023 in *Nature Communications* [4], Piña et al. identified and spatiotemporally localized increases in osteoblast differentiation gene markers and the introduction of terminally differentiated osteocytes within the palatal mesenchyme of Pax9 WT murine embryos during the developmental timepoints associated with palatal fusion. In this study, the osteogenic basis of palatal fusion was explored using bulk, single-nucleus, and spatially resolved RNA sequencing analyses, offering an increasingly resolved lens with which to view the gene expression patterns of the developing palate.

Bulk RNA-seq: To begin our transcriptomic analysis of the palatal shelves, total RNA was isolated from WT murine palatal shelves at both pre-fusion and post-fusion developmental ages and bulk RNA sequencing (bulk RNA-seq) was performed. Deploying bulk RNA-seq at the beginning of this study was a logical first step, as it allowed us to leverage this technique’s exceptionally high throughput to garner a broad picture of the genetic signaling environment of the palatal shelves at both pre- and post-fusion timepoints. Through principal component analysis (PCA) and differential gene expression (DGE) analysis of our bulk RNA-seq data, we pinpointed isolated increases in gene markers indicative of osteoblast commitment and differentiation at E14.5 to E15.5, identifying these timepoints as the critical onset of osteogenic programming in the embryonic palatal shelves.

Single-Nucleus RNA-Seq: Next, to validate and further clarify the transcriptomic shift previously identified through our bulk RNA-seq experiments, we collected microdissected palatal tissues from WT E13.5 and E15.5 murine embryos and deployed single-nucleus RNA sequencing (snRNA-seq). snRNA-seq represents a step up in resolution relative to bulk RNA-seq, specifically by providing information about distinct cell types and gene expression at a single-cell level. Through single-nucleus gene and transposase-accessible chromatin sequencing (snRNA+ATAC-seq) followed by gene ontology enrichment, we identified increases in bone marker expression profiles at E15.5 that were absent in the palatal tissues at E13.5, leading us to believe that the initial osteogenic transcriptional enrichment we identified through bulk RNA-seq was in fact correct.

Spatially Resolved (SP) RNA-Seq: While bulk and single-nucleus RNA-seq approaches are valuable in that they can provide meaningful global temporal transcriptomic data, these approaches fail to offer tissue-specific, spatial information critical to inferring genetic regulatory networks in vivo. Thus, to elegantly conclude our interrogation of the osteogenic basis of palatal fusion, we deployed two spatially resolved RNA sequencing approaches. First, coronal sections of E14.5 and E15.5 WT murine embryo heads were collected and the in situ mRNA hybridization (RNAscope, Advanced Cell Diagnostics) of select osteogenic markers was performed. Although primarily a qualitative assay, the in situ hybridization experiments provided a first glimpse into the spatiotemporal context of our discoveries. Then, to generate a more complete transcriptomic picture of the developing secondary palate—one that is contextualized and enhanced by spatiotemporal data—we collected mid-palatal sections of WT E14.5 and E15.5 embryos for transcriptomic comparison using the Visium platform by 10X Genomics, Inc. The Visium platform is a particularly valuable transcriptomic approach because it combines the per-cell biological insight of scRNA-seq with spatiotemporal gene expression patterns. Through our Visium experiment, we first confirmed the upregulation of key osteogenic gene markers from E14.5 (pre-fusion) to E15.5 (post fusion). To further address the hypothesis that we derived from prior sequencing studies, the temporal and spatial patterns of osteogenic markers and the presence of terminally differentiated osteocytes within the palatal mesenchyme were analyzed.

As evidenced by our study of the osteogenic basis of palatal fusion, RNA sequencing analyses can be deployed at differing levels of resolution to glean specific information from biological samples. In this study, we deployed concentrically narrowing RNA sequencing analyses, beginning with bulk RNA-seq (lowest resolution) and ending with spatially resolved RNA-seq (highest resolution), sequentially uncovering and then validating each new discovery along the way. Ultimately, this study provides a relevant scaffold for how multiple modes of transcriptomic analyses can be interwoven to create a cohesive set of observations that bear strong biological relevance.

By contrast, Xenium In Situ utilizes a microscopy-based approach to provide an ultra-high plex in situ assay with exceptional spatial resolution. For example, in a study published in the *Journal of Dental Research* in 2024 [9], “Spatial Multi-omics Reveals the Role of the Wnt Modulator, Dkk2, in Palatogenesis”, we describe the use of the Xenium In Situ platform—using a custom-designed panel of 350 palate-specific gene markers—to identify and spatially resolve the transcription of Dkk1 and Dkk2 to the osteogenic zones and mesial border within the midline of palatine bones in the murine Pax9^−/−^ cleft palate, respectively.

In this continuation study utilizing spatial transcriptomics to study palate development—now, with the use of a well-known disease model for palatal clefts—we utilized single-nucleus RNA + ATAC sequencing and spatially resolved RNA sequencing analyses. This allowed us to expand on the contemporary understanding of the functional roles of Pax9 and its associated Wnt modulators that bring about the osteogenic patterning and differentiation of the secondary palate.

Single-Nucleus (SN) RNA-Seq: First, the datasets produced in the previous study using microdissected Pax9 WT E13.5 and E15.5 murine palatal tissue were further assessed for cell-type-specific and binding motif enrichment. Through the initial clustering and gene ontology enrichment analysis of marker genes, cell types were identified and then clarified further through isolated analysis of the palatal mesenchyme. When compared to all cell types in the palate, our results indicated that Pax9+ mesenchymal cells (one of the subtypes we identified) demonstrated orders of magnitude greater Pax9 expression. Moreover, we noted a bias for the Pax9 motif within regions of accessible chromatin in the Pax9+ mesenchymal cells; meanwhile, however, osteogenic cells demonstrated depletion of the Pax9 motif and minimal Pax9 expression overall. Thus, we hypothesized that Pax9 may exert its influence on adjacent osteogenic cells through paracrine effects under the careful modulation of the Wnt signaling pathway.

Next, palatal tissue from Pax9^−/−^ E13.5 murine embryos was microdissected and processed for direct comparison with the previously generated snRNA+ATAC-seq dataset of normal secondary palate development at the same timepoint. We found that the E13.5 Pax9^−/−^ datasets displayed a relatively greater proportion of early and late osteoprogenitor cells compared to WT samples. We believed that this alteration in the palatal ossification process may be connected to the expanded expression domain of key Wnt signaling molecules Dkk1 and Dkk2, which were found to be increased in the Pax9 mutant tissue samples.

Spatially Resolved (SP) RNA-Seq: Building upon the previous RNAseq analysis, we performed the first application of spatial transcriptomic technology to dissect differential cell types and effector–ligand signaling interactions within the secondary palate both with and without the presence of functional Pax9. After custom designing a panel of 350 unique biomarker probes, we collected E14.5 Pax9^−/−^ and WT whole embryonic heads for analysis using the Xenium In Situ platform (10X Genomics, Inc.). UMAP plots were produced based on clusters of enriched genes and palate cell types were defined using these clusters. Our in situ analysis identified specific cluster and gene changes between Pax9^−/−^ and WT samples, indicating Wnt signaling dysregulation across cell types. Finally, using the Xenium In Situ platform, we validated our observations from the snRNA+ATAC-seq analysis, which suggested an expanded expression domain of key Wnt signaling molecules Dkk1 and Dkk2, by spatially resolving their transcription to the osteogenic zones and mesial border of the midline of osteogenic extension in palatine bone, respectively.

Our group’s most recent work [14] described the use of both Visium HD and Xenium In Situ assays for E12.5 and E13.5 WT and Pax9^−/−^ cleft palate murine embryos and developed a custom computational pipeline for refined oral versus nasal subregional analysis to highlight Pax9’s upstream regulatory role as a patterning transcription factor beyond the development of palatal bone, including other mesenchyme-derived cell types and extracellular components.

Unsurprisingly, ours is not the only research group exploring the development of the craniofacial tissues using spatial transcriptomic platforms. While our studies thus far highlight a relevant framework for deploying Xenium In Situ and Visium/Visium HD platforms for the investigation of palate and tooth development, other groups have demonstrated the ability to perform comparable analyses using different spatial platforms, such as seqFISH, stereoseq, Novaseq^TM^ 6000, and others [15,16,17]. Feng et al., for example, utilized scRNA-seq with Seurat v5 integration alongside a seqFISH-based spatial platform (Spatial Genomics) to develop a spatially resolved transcriptional map of the palatal mesenchyme in murine models at E12.5, E13.5, E14.5, E15.5, and E18.5. Interestingly, Feng et al. noted that, “while our focus here was primarily on the palatal mesenchyme, these techniques are readily applicable to other craniofacial mesenchymal tissues”, pointing toward the robustness and versatility of spatial-transcriptomic-based approaches across tissue types.

## 4. Spatial Transcriptomics to Study Tooth Development

Similar to palatogenesis, tooth development (odontogenesis) is a complex and dynamic process that progresses through the bud, cap, and bell stages, followed by root formation and eventual tooth eruption [18]. It is widely recognized that the Wnt signaling pathway is crucial for tooth development [19,20,21]. Wnt signaling molecules are activated in a spatiotemporal manner during odontogenesis, highlighting their significance in this process [22]. Mutations in Wnt signaling genes can lead to tooth agenesis and, among these WNT family members, WNT10A is the most frequently mutated gene in the non-syndromic selective agenesis of permanent teeth in humans. Studies on its role in mediating tooth epithelial–mesenchymal signaling are hence critical to our understanding of normal and abnormal tooth development. Individuals with WNT10A mutations present with diverse developmental dental anomalies, including microdontia of primary teeth, impaired roots, and cusp formation, and the complete absence of permanent dentition as seen in select forms of ectodermal dysplasia [23].

Our analyses revealed an intense expression of *Wnt10a* in both pre-differentiated and fully matured odontoblasts, with levels persisting in odontoblasts near Hertwig’s epithelial root sheath (HERS) [22]. This offers an explanation for why a deficiency in Wnt10a results in enamel hypoplasia, contributing to a flattened crown and taurodontic root formation in both mice and humans [24]. The secreted proteins Dickkopf (Dkk) and Sclerostin (Sost) function as Wnt inhibitors by preventing Wnt ligands from binding to Lrp 5/6 receptors [25]. Blocking DKK1 and SOST with a bispecific antibody has demonstrated enhanced bone repair efficacy compared to monotherapy. Targeting key regulators of the Wnt signaling pathway presents significant potential for tooth regeneration [26]. Thus, a thorough understanding of their spatiotemporal expression patterns during odontogenesis in embryonic and postnatal development is essential. Our team conducted a spatiotemporal analysis of Wnt mediators, such as *Sost* and *Dkk1*, along with the Wnt effector ligand *Wnt10a*, during odontogenesis, utilizing advanced multiplex technology with high spatial resolution as described earlier [22].

**Figure 2:**
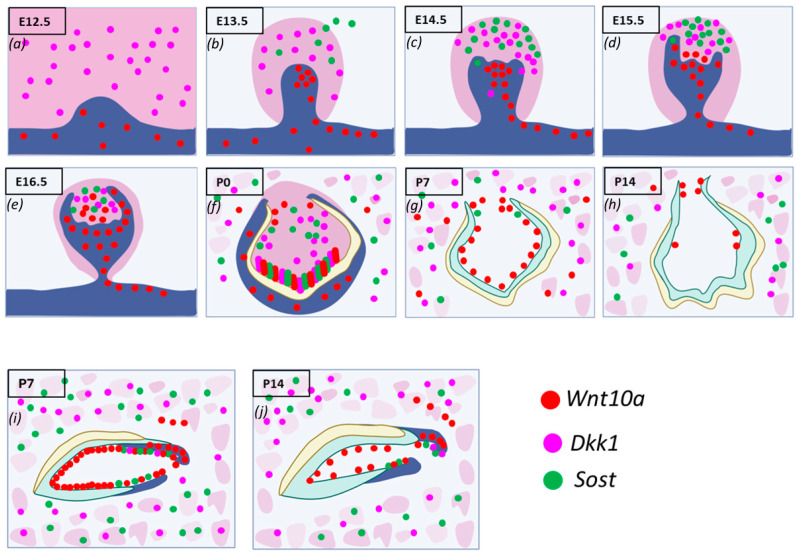
Schematic representations of spatiotemporal expression patterns of *Wnt10a*, *Dkk1*, and *Sost* with expressions in red dots, pink dots, and green dots, respectively, on coronal sections of the maxillary first molar and sagittal sections of the maxillary incisor tooth organ of wild-type mice in different developmental stages. The major expression signals are summarized here: (**a**) mesenchyme; (**b**) epithelial invagination; (**c**) dental papilla mesenchyme; (**d**) palatal cervical loop; (**e**) patchy expression in dental papilla mesenchyme; (**f**) terminally differentiating and secretory odontoblasts; (**g**) Hertwig’s epithelial root sheath; (**h**) weak in odontoblasts; (**i**) intense in odontoblasts; (**j**) intense in ameloblasts and odontoblasts.

During the initiation of tooth development, *Wnt10a* transcripts were detected in the oral and presumptive dental epithelium but not in the mesenchyme of maxillary and mandibular molars. *Dkk1* was expressed in the mesenchyme of molars, while *Sost* expression was minimal or absent (Figure 2a). Similar patterns were found in the incisors. By E13.5, *Wnt10a* transcripts were found throughout the molar bud epithelium, with the highest expression in the enamel knot (EK). The strongest expression in the dental epithelium was observed on the buccal side, while *Sost* and *Dkk1* were found within the mesenchyme. *Wnt10a* was also strongly expressed along the labial border of the epithelial invagination in incisors (Figure 2b). In the cap stage (E14.5), the EK structure was well-defined in molars. *Wnt10a* was localized exclusively to the EK area, with slightly stronger signals on the buccal side. Sost and Dkk1 showed enriched expression in the dental papilla (DP) mesenchyme, absent from the coronal papilla adjacent to the EK (Figure 2c).

By E15.5, molars and incisors began to progress to the bell stage. *Wnt10a* expression started shifting to the surrounding mesenchymal cells adjacent to the EK, with diffuse expression in the EK region and intense expression in the buccal dental epithelium of molars. *Sost* and *Dkk1* transcripts remained unchanged from E14.5, with a concurrent expression of both Wnt modulators absent in the coronal papilla near the EK. A weaker expression of *Sost* and *Dkk1* was observed around the longer palatal cervical loop (CL) compared to the shorter buccal side of the molars (Figure 2d). In incisors, both *Sost* and *Dkk1* were expressed in the mesenchyme closer to the EK region [22].

Sagittal sections of molars at E16.5 and E17.5 revealed patchy expression patterns of Wnt mediators in the DP mesenchyme, with an absence in areas of intense Wnt10a transcripts (Figure 2e). At P0, secretory odontoblasts were aligned perpendicular to the basement membrane, with predentin deposition evident in mandibular molars. Enamel formation was visible at the labial surface of the mandibular incisors. *Sost* and *Dkk1* were strongly localized along the secretory odontoblast layer of molars and incisors. *Wnt10a* was intensely expressed in terminally differentiated ameloblasts and odontoblasts at the developing cusp tips of molars and incisors, with weaker expression observed in the ameloblasts at the occlusal groove of mandibular molars. A reduced expression of *Wnt10a*, *Sost*, and *Dkk1* was seen in odontoblasts at the occlusal groove. This multiplex in situ analysis at P0 suggests a colocalization pattern of *Wnt10a*, *Dkk1*, and *Sost* in terminally differentiating and secretory odontoblasts (Figure 2f).

*Dkk1* and *Sost* generally showed overlapping expression patterns, with a close spatial association throughout development from E14.5 to P0. *Wnt10a* transcripts were localized to a single odontoblast layer, and the expression of Wnt modulators decreased following the mineralization of enamel and dentin. By P7, the advanced mineralization of enamel and dentin occurred in molars and incisors. *Wnt10a* transcripts were primarily localized to odontoblasts, with a weaker signal in the reduced dental epithelium (RDE). Elongated HERS in molars showed a strong *Wnt10a* signal. *Sost* expression was lower and exclusively present in certain areas of the RDE, with a few punctate signals of *Sost* and *Dkk1* in odontoblasts near the molar HERS (Figure 2g).

Maxillary incisors showed strong *Wnt10a* expression in ameloblasts and odontoblasts near the proximal end of the CL. Intense signals of *Sost* and *Dkk1* were also observed in the odontoblast layer of maxillary incisors at P7 (Figure 2i). At P14, weak *Wnt10a* expression was seen in odontoblasts, with no expression of *Sost* or *Dkk1* in molars (Figure 2h). Intense *Wnt10a* signals were continuously present in ameloblasts and odontoblasts near the proximal end of the incisors at P14, with abundant *Sost* and *Dkk1* transcripts observed in proximal odontoblasts near the CL in incisors (Figure 2j). The enriched expression of *Sost* and *Dkk1* was also detected along the future alveolar bone regions surrounding the tooth organ from E12.5 to E15.5, with intense expression in osteocytes of developing bone during later stages (Figure 2a–j). A detailed spatiotemporal analysis of Wnt ligand and modulator expression during tooth development provided new insights into the essential regulation of Wnt signaling required for proper cellular differentiation, patterning, and homeostasis.

While progress in technological advancement and scientific insight in spatial transcriptomics have grown exponentially in recent years, there still remain some critical limitations to its use for genetic mapping. The two-dimensional nature of histological sections remains a barrier to studying networks of cells and tissues in situ as they extend in three dimensions. Relatively small areas of capture fitted for tissue placement on spatial assays restrict not only the type and size of tissue samples analyzed but also the statistical power of potential analyses to be performed. As such, the ability to scale spatial omics approaches to the same degree as non-spatial single-cell assays remains limited due to exorbitant costs and technical constraints. Furthermore, the breadth of gene coverage (while in the process of expansion) is still limited to known genes, limiting the extent of discovery-based research possible through such assays. In such a rapidly evolving field, there is hope that these limitations and others not mentioned will be resolved and refined for optimal spatial analyses.

As we look to the future of applied spatial omics technologies toward diagnostic and therapeutic innovation for anomalies of the craniofacial complex, there is tremendous potential. As seen in our preclinical studies to date, these technologies have the capacity to resolve single molecules in their native expression environment, pinpointing biological function during development. As clinical protocols evolve to more patient-centered, precision-based strategies, such technologies could be vital in properly diagnosing and treating complex genetic anomalies in children and adults. We hope the field of spatial omics—alongside precision tissue engineering and regeneration—will continue to expand in clinical application, potentially offering greater translational options for patients from bench-to-bedside.

## Data Availability

The original work cited in this review article can be accessed openly at the following links: https://www.nature.com/articles/s41467-023-41349-9 (accessed on 1 April 2025); https://journals.sagepub.com/doi/full/10.1177/00220345241256600 (accessed on 3 April 2025); https://www.researchsquare.com/article/rs-5969552/v1 (accessed on 5 April 2025); https://www.frontiersin.org/journals/physiology/articles/10.3389/fphys.2023.1316635/full (accessed on 5 April 2025).
